# Creative Aging Literature Review: The Role and Efficacy of Arts-Based Interventions in Older Adult Populations

**DOI:** 10.1093/geroni/igaf122.2135

**Published:** 2025-12-31

**Authors:** Eileen Tell, Jacob Watson

**Affiliations:** ET Consulting, LLC, Bemont, Massachusetts, United States; The Practice, Chicago, Illinois, United States

## Abstract

The term “creative aging” comes from a 2006 landmark study by Dr. Gene Cohen (The Creativity and Aging Study) which evidenced the promise for arts-based learning to support overall health and wellness of older adults. As lifespans increase, there is a need for interventions that sustain the well-being of older adults living longer lives. This presentation reviews the relevant literature and offers an environmental scan of best practices in creative aging to provide design considerations and practical recommendations for those interested in bring creative aging initiatives into their practice. Specifically, we explore prior research across various art forms and the outcomes identified regarding: cognitive and physical health; mental and emotional well-being; and health care spending and/or utilization. The literature review summarizes measurement practices, study designs, research limitations, and key findings. The environmental scan describes the variety of sites and program types where creative aging takes place including: senior/assisted living communities; arts-based organizations (museums, theatres, galleries) and public and community spaces. We present evidence that participant voice and choice, cultural relevance and quality of instruction have a critical impact on the success of the intervention and the social, emotional, and cognitive benefits to participants than any single medium or art form. We conclude with considerations for program design and challenges regarding replicability and bringing programs to scale, such as duration and dosage, choice of art form, active vs. passive participation, instruction quality, and research design.

